# Severe Fever with Thrombocytopenia Syndrome Virus Infection, South Korea, 2010

**DOI:** 10.3201/eid2411.170756

**Published:** 2018-11

**Authors:** Young Ree Kim, Yeojun Yun, Seung Geon Bae, Dahee Park, Suhyun Kim, Jae Myun Lee, Nam-Hyuk Cho, Yang Soo Kim, Keun Hwa Lee

**Affiliations:** Jeju National University College of Medicine, Jeju, South Korea (Y.R. Kim, S.G. Bae, D. Park, S. Kim, K.H. Lee);; Ewha Womans University, Seoul, South Korea (Y. Yun);; Yonsei University College of Medicine, Seoul (J.M. Lee);; Seoul National University College of Medicine, Seoul (N.-H. Cho);; University of Ulsan College of Medicine, Seoul (Y.S. Kim).

**Keywords:** severe fever with thrombocytopenia virus, thrombocytopenia, South Korea, viruses, SFTSV

## Abstract

Severe fever with thrombocytopenia syndrome (SFTS) was reported in China in 2009 and in South Korea in 2012. We found retrospective evidence of SFTS virus infection in South Korea in 2010, suggesting that infections in South Korea occurred before previously reported and were more concurrent with those in China.

Severe fever with thrombocytopenia syndrome virus (SFTSV) is a tickborne virus (genus *Phlebovirus*, family *Phenuiviridae*) that can cause hemorrhagic fever (*1*,*2*). Severe fever with thrombocytopenia syndrome (SFTS) was confirmed in China in 2009 (*2*) and then retrospectively reported in South Korea in 2012 and in western Japan in 2013 (*3*,*4*). Most SFTSV infections occur through bites from *Haemaphysalis longicornis* ticks, although transmission can also occur through close contact with an infected patient (*5*–*7*). We provide retrospective evidence of SFTSV infections in South Korea from 2010, confirming that infections in South Korea occurred earlier than previously reported and were more concurrent with the first reported infections in China.

For this study, we used stored serum samples from 58 patients who had high erythrocyte sedimentation rates (ESRs) and were admitted to Jeju National University Hospital in Jeju, South Korea, during July 2010; SFTS was not a criterion for selection. Jeju is a high-prevalence region for SFTS, and July is the month with the highest SFTS prevalence in South Korea. The major clinical signs and symptoms of SFTS are an acute and high fever, thrombocytopenia, leukopenia, elevated serum hepatic enzyme levels, gastrointestinal symptoms, and multiorgan failure, with a death rate of 12%–30%. However, atypical signs and symptoms of SFTS have been identified, and asymptomatic infections among humans have been reported in South Korea (*3*,*6*,*8*).

For molecular diagnosis of SFTSV, we extracted RNA from serum by using the QIAamp Viral RNA Mini Kit (QIAGEN, Hilden, Germany). We performed real-time reverse transcription PCR (RT-PCR) to amplify the partial small (S) segment of the viral RNA from the stored serum and confirm SFTSV infection (*9*). We sequenced real-time RT-PCR products using the BigDye Terminator Cycle Sequencing kit (Perkin Elmer Applied Biosystems, Warrington, UK). We conducted phylogenetic analysis of partial S segment sequences using MEGA6 (*10*) and constructed phylogenetic trees using the maximum-likelihood method (Figure).

**Figure:**
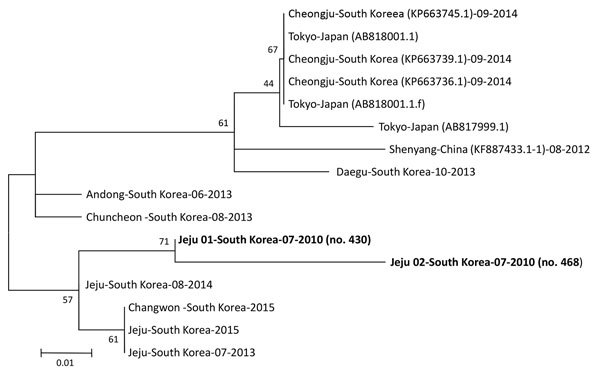
Phylogenetic tree constructed based on partial small segment sequences of severe fever with thrombocytopenia syndrome virus identified in stored serum samples collected in 2010 from 2 patients in South Korea (bold) compared with reference viruses. We constructed the tree using the maximum-likelihood method with MEGA 6 (*10*). The partial small sequence data for the viruses identified in China, South Korea, and Japan were obtained from GenBank (accession numbers in parentheses). Scale bar indicates nucleotide substitutions per site.

Our results showed 2 positive results from the stored serum of 2 patients. Neither patient reported history of travel to other endemic countries, such as China or Japan.

Patient Jeju 01-South Korea-07-2010 (no. 430), a 77-year-old man, had diabetes and hypertension for 45 years. The patient had received a diagnosis of end-stage renal disease 7 years before admission and was on hemodialysis; he experienced an episode of gout 5 years before admission. The patient came to the hospital’s orthopedic outpatient clinic reporting that he had pain, swelling, and febrile sensation of the right lateral malleolus area for several days. He had undergone orthopedic surgery with debridement for a wound of the right lateral malleolus area caused by an electric plate burn 1.5 years earlier. He was retired, lived in Jeju, and was admitted to the hospital for emergency orthopedic surgery. Physical examination recorded body temperature of 37.2°C, blood pressure 140/60 mm Hg, heart rate 78/min, and respiratory rate 20 breaths/min. Initial laboratory data revealed slight thrombocytopenia (199,000 platelets/mm^3^), and absolute neutrophil count (ANC) was 7,374. Alkaline phosphatase (ALP), aspartate aminotransferase (AST), and alanine aminotransferase (ALT) levels were within reference ranges; however, ESR (112 mm/h), blood urea nitrogen (24.8 mg/dL), and creatinine (4.8 mg/dL) were elevated.

Patient Jeju 02-South Korea-07-2010 (no. 468), a 76-year-old woman, visited the hospital’s orthopedic outpatient clinic reporting pain in both knees for 10 years. She was a housewife, lived in Jeju, and had received temporary symptom treatment from local clinics. She was admitted to the hospital for total knee replacement arthroplasty. Her only underlying disease was diabetes. Physical examination recorded body temperature of 36.8°C, blood pressure 120/60 mm Hg, heart rate 68/min, and respiratory rate 20 breaths/minute. Initial laboratory test results were within reference ranges except for urine glucose (4+); platelet count was 620,000/mm^3^, ANC was 2,065, and ALP, AST, and ALT levels were within reference ranges. Her ESR level was elevated, at 74 mm/h.

SFTSV was reported in South Korea in 2012 (*3*). In this study, we retrospectively confirmed 2 SFTSV infections in South Korea in 2010 by amplification of the partial S segment of the viral RNA from stored serum of patients with a high ESR. Atypical signs and symptoms of SFTS among patients and asymptomatic infections have previously been reported in South Korea (*3*,*6*,*8*). In this study, we found that the signs and symptoms of SFTS were distinct from the major clinical signs and symptoms of SFTS, and the knee pain was coincidence. However, a case study showed that 1 SFTS patient had a crusted erythematous ulcer on the lateral side of the left knee, accompanied with left inguinal lymphadenopathy.

In conclusion, we suggest that SFTSV infections in South Korea have occurred during a period similar to the period reported for China, where SFTSV was found in 2009. In addition, the signs and symptoms of SFTS may be atypical. Therefore, further clinical, epidemiologic, and laboratory research is needed to better understand the transmission dynamics of SFTSV and prevent additional SFTSV infections in other populations.
